# Phenotypic and Proteomic Characteristics of Human Dental Pulp Derived Mesenchymal Stem Cells from a Natal, an Exfoliated Deciduous, and an Impacted Third Molar Tooth

**DOI:** 10.1155/2014/457059

**Published:** 2014-10-14

**Authors:** Gurler Akpinar, Murat Kasap, Ayca Aksoy, Gokhan Duruksu, Gulcin Gacar, Erdal Karaoz

**Affiliations:** ^1^DEKART Proteomics Laboratory, Kocaeli University Medical School, Umuttepe, 41380 Kocaeli, Turkey; ^2^Department of Medical Biology, Kocaeli University Medical School, Umuttepe, 41380 Kocaeli, Turkey; ^3^Department of Stem Cell, Center for Stem Cell and Gene Therapies Research and Practice, Institute of Health Sciences, Umuttepe, 41380 Kocaeli, Turkey; ^4^Liv Hospital, Center for Regenerative Medicine and Stem Cell Research & Manufacturing (Liv MedCell), Besiktas, 34340 Istanbul, Turkey

## Abstract

The level of heterogeneity among the isolated stem cells makes them less valuable for clinical use. The purpose of this study was to understand the level of heterogeneity among human dental pulp derived mesenchymal stem cells by using basic cell biology and proteomic approaches. The cells were isolated from a natal (NDPSCs), an exfoliated deciduous (stem cells from human exfoliated deciduous (SHED)), and an impacted third molar (DPSCs) tooth of three different donors. All three stem cells displayed similar features related to morphology, proliferation rates, expression of various cell surface markers, and differentiation potentials into adipocytes, osteocytes, and chondrocytes. Furthermore, using 2DE approach coupled with MALDI-TOF/TOF, we have generated a common 2DE profile for all three stem cells. We found that 62.3 ± 7% of the protein spots were conserved among the three mesenchymal stem cell lines. Sixty-one of these conserved spots were identified by MALDI-TOF/TOF analysis. Classification of the identified proteins based on biological function revealed that structurally important proteins and proteins that are involved in protein folding machinery are predominantly expressed by all three stem cell lines. Some of these proteins may hold importance in understanding specific properties of human dental pulp derived mesenchymal stem cells.

## 1. Introduction

Stem cells are undifferentiated cells that can divide, differentiate, and self-renew to produce new stem cells in multicellular organisms [1]. They can be used in biomedical research, drug discovery, and toxicity testing, as a model in understanding diseases and more importantly for therapeutic purposes in regenerative medicine [2]. To use stem cells successfully in the aforementioned areas, homogenous populations of stem cells have to be isolated, identified, and characterized. However, given the extent of heterogeneity within and among the stem cell lines, the isolation of homogenous stem cell populations appears to be a challenging task [3].

Although there is a descriptive definition for mesenchymal stem cells (MSCs), the extent of heterogeneity within and among MSC lines is overwhelming [4]. This creates a lack of extensive overlap among the studies performed with MSCs. In addition to the genetic background, methods of derivation, growth conditions, the stage of the cell cycle during sample collection, the age and gender of the donor, and the disease status of the donor are the likely factors that contribute to the heterogeneity problem [5]. In general, characterization of MSCs heavily relies on the use of methods such as immunofluorescence microscopy, reverse transcription PCR, and flow cytometry to establish both stem cell identity and function. However, to facilitate stem cell definition through cellular phenotypic profile, comparative analysis of gene and protein expression studies should be performed. Currently there is no universally accepted and commonly used cellular phenotypic profile for stem cell characterization. Gene expression profiles are preferred due to their relative ease but they vary greatly with the organisms' state and environment in ways that cannot be easily interpreted. The signature obtained from analysis of the total cell proteome or cell surface proteome (“protein barcodes”) is promising and proteomic approaches can be powerful in characterizing the entire protein profile of stem cell phenotype from different niches.

To understand the level of heterogeneity among the MSCs, we isolated MSCs from dental pulps of a natal, an exfoliated deciduous, and an impacted third molar tooth of three different donors. The isolated stem cells were then cultured under the same growth conditions and passaged similarly. The cells were compared on the basis of cellular morphology and expression of MSC specific markers and pluripotent transcription factors. In addition, telomerase activity measurements were performed to collect information about age related changes and cellular senescence. Finally, we compared the protein expression profiles of undifferentiated cells by using 2DE gel electrophoresis followed by MALDI-TOF/TOF MS/MS analysis. We identified 61 proteins that were predominantly expressed by all three stem cell lines. We believe that some of these proteins may hold importance in understanding specific properties of human dental pulp derived mesenchymal stem cells.

## 2. Materials and Methods

### 2.1. Isolation and Culture of MSCs from Human Dental Pulps (Natal, Deciduous, and Third Molar)

Isolation and culture of human dental pulp derived MSCs were performed according to protocols described elsewhere [6]. Briefly, dental pulps of exfoliated deciduous and impacted third molar teeth were collected by cutting around the cement-enamel junction by using sterilized dental fissure burs to reveal the pulp chamber. The recovery of natal dental pulp is different and harder compared to pulp from adult teeth, where teeth were cut around the cementoenamel junction using dental fissure burs to open the pulp chamber and separate the pulp tissue from the crown and root by an excavator. In the recovery of natal teeth pulp, pliers were used to fracture the dental crown into several parts and the dental pulp was uncovered. The pulp tissue of each sample was gently separated from the crown and root by using sterile excavator and digested with collagenase type I to generate single cell suspensions. MEM-Earle medium containing 15% fetal bovine serum and 100 IU/mL penicillin-100 *μ*g/mL streptomycin was used and the cells were cultured in 25 cm^2^ plastic tissue culture flasks, incubated at 37°C in a humidified atmosphere containing 5% CO_2_. The stem cells were isolated on the basis of their ability to adhere to the culture plates. On the third day, red blood and other nonadherent cells were removed and the medium was replaced to allow further growth. The adherent cells grown to 70% confluency were defined as passage zero (P_0_) cells. Later passages were labeled accordingly. 0.25% trypsin-EDTA solution was used to detach the cells from the plate for passaging and the detached cells were centrifuged at 2000 ×g for 10 minutes, resuspended in one mL complete medium, counted twice using a Thoma counting chamber, and then plated in 75 cm^2^ flasks (BD Biosciences, USA) at densities of 1 × 10^6^ cell/flask. Growth medium was replaced every three days over a 10–14-day period.

### 2.2. Characterization of MSCs by Flow Cytometry

To confirm that MSCs maintain their phenotypic characteristics* in vitro*, undifferentiated cells were subjected to flow cytometry analyses. After passage three, stem cells were harvested and resuspended in culture medium at a concentration of 10^6^ cells/mL. Flow cytometry was performed by using FACSCalibur (BD Biosciences, USA) according to Karaöz et al. (2010) [6]. The data were analyzed with CellQuest software (BD Biosciences, USA).

Immunophenotyping characterization of MSCs was performed with antibodies against the following human antigens: CD3, CD4, CD13, CD14, CD29, CD34, CD44, CD45, CD73, CD90, CD106, CD117, CD146, CD166, HLA-DR, and HLA-ABC (Table 1). The antigen selection was made based on MSC defining criteria set by Dominici et al. (2006) [7]. All antibodies were from BD Biosciences. More than 50% staining was regarded as positive.

### 2.3. *In Vitro* Stem Cell Differentiation

To induce adipogenic differentiation 3000 cells/cm^2^ were seeded onto six-well plates and cultured with Mesencult MSC Basal Medium supplemented with 10% adipogenic supplement (StemCell Technologies Inc., Canada) and 1% penicillin/streptomycin for three weeks. The medium was refreshed every two days. Intracellular lipid droplets indicating adipogenic differentiation were confirmed by Oil Red O staining.

For osteogenic differentiation, 3000 cells/cm^2^ were seeded onto collagen (type I) coated cover-slips in six-well plates. The differentiation medium was changed twice a week. After four weeks, osteogenic differentiation was assessed by Alizarin Red staining.

To induce chondrogenic differentiation, pelleted micro-mass of 2.5 × 10^5^ cells was formed by centrifugation at 1300 ×g for 5 minutes and then cultured with chondrogenic medium for two weeks. Medium was changed twice a week. The pellets, fixed with 4% paraformaldehyde and embedded into paraffin, were stained with Haematoxylin-Eosin and Safranin O to assess chondrogenic differentiation.

### 2.4. Measurement of Telomerase Activity

Telomerase activity was measured by conventional telomeric repeat amplification protocol (TRAP) using TeloTAGGG PCR-ELISA kit (Roche Diagnostics, Germany) according to the manufacturer instructions. In brief, after lysis of 2 × 10^5^ cells, three *μ*L of supernatant was incubated with premixed buffer for 20 min at 25°C to show the presumed telomerase activity, and the products were amplified with biotin-labeled primers by PCR. Streptavidin coated ELISA plates were used to quantify the activity by addition of peroxidase specific substrate (3,3′,5,5′-tetramethylbenzidine dihydrochloride; TMB) and reading the optical density at 450 nm. Relative telomerase activity (RTA) was calculated with respect to the control template equivalent to 0.001 mol/*μ*L DNA.

### 2.5. Cell Cycle Analysis

Cell cycle was determined with flow cytometry by using BD Cycletest Plus kit (BD Biosciences, USA) following the protocol provided by the manufacturer.

### 2.6. Protein Extraction and 2DE Gel Electrophoresis

For analysis of proteome profiles, cells were grown in defined media (DMEM, low glucose, pyruvate, Life Tech. Inc., USA). To start cultures, equal number of cells (3 × 10^5^) was seeded into T-75 flasks in triplicate. When 70% confluency was reached the cells were washed with ample amount of ice-cold wash buffer (10 mM Tris-HCl, pH 7.0, 250 mM sucrose) for three times and removed from the plates by scraping. After 10 min centrifugation at 4°C at 2000 ×g, excess wash buffer was decanted and 250 *μ*L of cell lysis buffer 2DE rehydration buffer (8 M urea, 2 M thiourea, 4% CHAPS, 30 mM Tris pH 8.5, and 1x protease inhibitor cocktail) was added over each cell pellet. To achieve complete lysis, cells were vigorously vortexed for 1 min in lysis buffer and the supernatant containing the soluble protein fraction was obtained by centrifugation at 20.000 ×g for 30 min at 4°C and stored in lobind tubes (Eppendorf, USA) at −80°C after being snap-frozen in liquid nitrogen. Protein concentration was determined by using modified Lowry assay with the BSA standard (Bio-Rad, USA). 80 *μ*g of protein was loaded onto immobilized pH gradient strips (IPG) (11 cm, pH 5–8) (Bio-Rad, USA) via passive rehydration. Separation based on isoelectric points was performed by using Protean isoelectric focusing cell using the recommended conditions (Bio-Rad, USA). A stepwise incremental voltage program was applied to each strip (250 V for 20 min (linear), 4000 V for 2 hr (linear), and 10000 V/hr (rapid)) by using Protean IEF system (Bio-Rad, USA). The strips were then subjected to a two-step equilibration in equilibration buffers containing 6 M urea, 2% SDS, 0.375 M Tris Cl pH 8.8, 20% glycerol, and 2% DTT for the first step and the same buffer without DTT but with iodoacetamide (2.5%) for the second step. Following isoelectric focusing, strips were subjected to SDS-PAGE. Precast gels (Criterion TGX Any kD, Bio-Rad, USA) were used with Criterion Dodeca gel running system (Bio-Rad, USA) to minimize gel to gel variation. Gels were stained with Colloidal Coomassie stain (KeraFast, USA) and visualized with VersaDoc4000 MP (Bio-Rad, USA). PDQuest Advance (Bio-Rad, USA) 2DE analysis software was used for comparison of protein spot profiles. For automated spot detection, parameters used were sensitivity (13.8), spot size scale (3), and minimum peak intensity (258).

### 2.7. Image Analysis and Spot Cutting

The outside edges of all of the images were identically cropped by using automated crop tool of PDQuest Advance software (Bio-Rad, USA). Stain speckles were filtered and the standardized areas of interest from all gels were matched and warped and the quantity of each spot was normalized by the total valid spot intensity. Total spot numbers and volumes within the normalized area were determined from the automated analyses. A manual editing tool was used to inspect the determined protein spots detected by the software. Spots that were prone to variation were excluded if they were hard to identify by visual inspection. Every-member matching protein spots were selected among the three mesenchymal stem cells lines. The spots were cut by using automated spot cutting tool, ExQuest spot cutter (Bio-Rad, USA), and disposed into 96-well plates for protein identification.

### 2.8. Identification of Proteins

Protein identification experiments were performed at Kocaeli University DEKART proteomics laboratory (http://kabiproteomics.kocaeli.edu.tr/) by using ABSCIEX MALDI-TOF/TOF 5800 system. In-gel tryptic digestion of the proteins was performed by using an in-gel digestion kit following the recommended protocol by the manufacturer (Pierce, USA). Before deposition onto a MALDI plate, all samples were desalted and concentrated with a 10 *μ*L ZipTip_C18_ following the recommended protocol (Millipore, USA). Peptides were eluted in a volume of 1 *μ*L using a concentrated solution of *α*-CHCA in 50% acetonitrile and 0.1% trifluoroacetic acid in water and spotted onto the MALDI target plate. The TOF spectra were recorded in the positive ion reflector mode with a mass range from 400 to 2000 Da. Each spectrum was the cumulative average of 200 laser shots. The spectra were calibrated with the trypsin autodigestion ion peaks* m/z* (842.510 and 2211.1046) as internal standards. Ten of the strongest peaks of the TOF spectra per sample were chosen for MS/MS analysis.

All of the PMFs were searched in the MASCOT version 2.5 (Matrix Science) by using a streamline software, ProteinPilot (ABSCIEX, USA), with the following criteria: National Center for Biotechnology Information nonredundant (NCBInr); species restriction to* H. sapiens;* enzyme of trypsin; at least five independent peptides matched; at most one missed cleavage site; MS tolerance set to ±50 ppm and MS/MS tolerance set to ±0.4 Da; fixed modification being carbamidomethyl (Cys) and variable modification being oxidation (Met); peptide charge of 1+ and being monoisotopic. Only significant hits, as defined by the MASCOT probability analysis (*P* < 0.05), were accepted (see Supplementary Material 1  in Supplementary Material available online at http://dx.doi.org/10.1155/2014/457059).

Classification of proteins was performed by using a freely available classification system PANTHER (http://www.pantherdb.org/) as well as NCBI (http://www.ncbi.nlm.nih.gov/pubmed)  and Swiss-Prot/TrEMBL annotations (http://www.expasy.org/) [8].

### 2.9. Validation of the Selected Proteins by Western Blot Analysis

The same protein preparations from the cells used in the 2DE gel experiments were used for Western blotting (WB) to validate the identified proteins by MALDI-TOF/TOF. Protein extracts (30 *μ*g protein for each lane) were mixed with SDS sample buffer (Laemmli solution) and they were subjected to 12% SDS-PAGE gels. The gels were transferred onto nitrocellulose membranes (0.45 *μ*m, Bio-Rad) at 30 mA constant current (TurboBlot, Bio-Rad). To prevent nonspecific bindings, the membranes were blocked for 1 h at room temperature in 5% nonfat dried milk, 1% (v/v) Tween 20 in TBS (50 mM Tris, 150 mM NaCl, adjust pH with HCl to pH 7.6). After blocking, the membranes were incubated overnight at 4°C with appropriately diluted polyclonal primary antibodies in TBS-T against Galectin-1 (1 : 2000, Santa Cruz, sc-28248), DJ-1 (1 : 1000, Abcam, ab-4150), HNRNPH1 (1 : 10000, Abcam, ab10374), GAPDH (1 : 2500, Abcam, ab-9485), and monoclonal UCHL-1 (1 : 5000, Pierce, MAI-46079). GAPDH was used for normalization of each protein sample to ensure equal protein loading. Following three washes with TBS-T, the membranes were incubated with the secondary anti-mouse IgG HRP antibody (Bio-Rad) or anti-rabbit IgG (Bio-Rad) for 1 h at room temperature. Then the proteins were visualized with an ECL Plus Western blotting detection system (GE Healthcare). The bands were quantitated by using Quantity One 1D image analysis software (Bio-Rad).

### 2.10. Statistical Analysis

Flow cytometry, stem cell differentiation and telomerase activity measurements were performed on passage three cells. Any data presented showing the results of a set of analysis were carried out on all cell types. The number of experimental replications, both technical and biological was kept at three.

When it was necessary, statistical analysis of data was carried out by one-way analysis of variance (ANOVA), followed by Tukey's test for multiple comparisons to determine the values that were significantly different. Differences were considered statistically significant at *P* < 0.05.

## 3. Results

**Figure 1:**
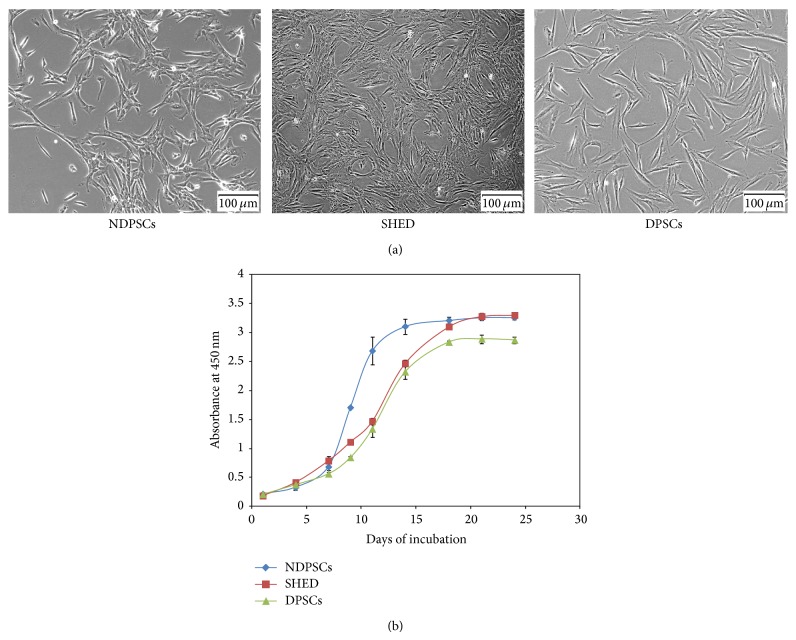
Illustration of MSC morphologies and relevant growth kinetics. (a) Morphology of NDPSCs, SHED, and DPSCs. The cells were at passage three. Images were taken with an inverted microscope (40X). (b) Growth curves for NDPSCs, SHED, and DPSCs over a period of 25 days. The cells were cultured in 96-well plates in triplicate and their growth rates were followed by WST-1 assay, which measures cell viability. DPSCs displayed relatively lower growth rate than SHED and NDPSCs.

MSC lines that were isolated and used throughout the study were from three different donors. The NDPSCs were isolated from dental pulp of a tooth that belonged to a female baby who was born with a tooth. The SHED was isolated from dental pulp of a female juvenile who lost one of her deciduous tooth at the age of six and the DPSCs were from a dental pulp of an impacted third molar tooth that belonged to a 27-year-old female. All three stem cell lines were characterized to some extent. Morphological analysis of each stem cell line indicated the presence of cells with large, flattened, or fibroblast-like shape (Figure 1(a)). No noteworthy changes in morphology and growth characteristics were observed throughout the passages. Analysis of the common MSC markers with flow cytometry indicated that CD13, CD29, CD44, CD73, CD90, CD146, CD166, and HLA-ABC were expressed by all three stem cell lines (Table 1). Evaluation of proliferation rates by measuring cell viability at various time points indicated that NDPSCs had higher proliferation rate than SHED and DPSCs (Figure 1(b)).

**Figure 2:**
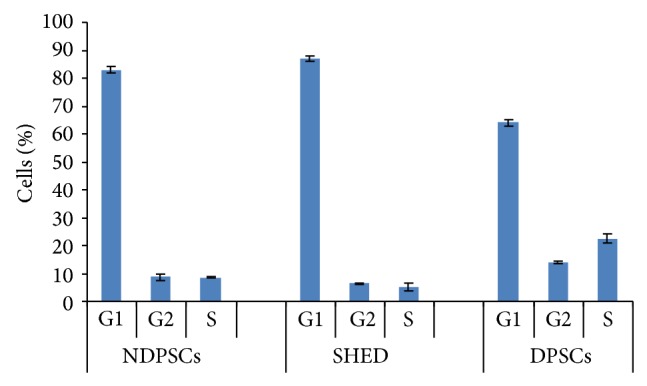
Cell cycle analysis of NDPSCs, SHED, and DPSCs. Each sample (10,000 cells) was counted in triplicate by a flow cytometer. The values are expressed as percentage mean ± standard deviation.

As analyzed by FACS, majority of the stem cells (78%) were in G1 phase of the cell cycle indicating that the cells were growing in size and synthesizing their mRNA and proteins (Figure 2). The cells that were in S and G2 phases were present in lower ratios (12% and 10%, resp.); thus the number of cells undergoing mitotic division was less than the actively growing cells.

**Figure 3:**
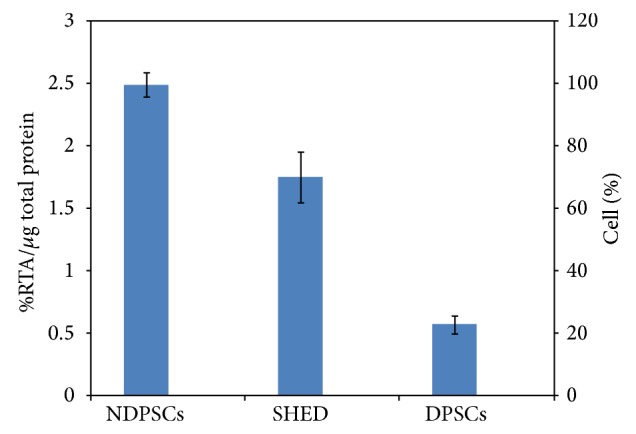
Telomerase activities of NDPSCs, SHED, and DPSCs. The activities were measured by conventional telomeric repeat amplification protocol (TRAP) and relative telomerase activities (RTA) were calculated with respect to the control template equivalent to 0.001 mol/*μ*L DNA.

Age related telomerase shortening due to loss of telomerase activity is an indicator of stem cell aging [9–11]. We observed a donor-age associated decrease in telomerase activities. The highest telomerase activity was observed with NDPSCs (Figure 3) (considered to be 100% in relative ratio). There was a 30% decrease in telomerase activity of SHED in comparison to NDPSCs. The lowest telomerase activity observed was in DPSCs.

**Figure 4:**
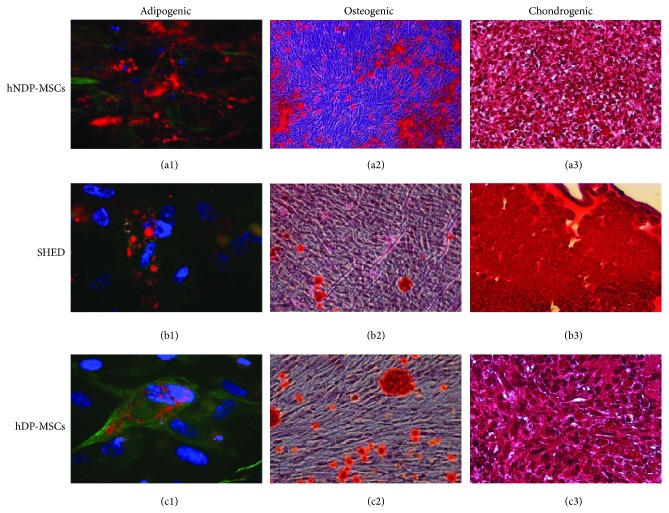
*In vitro* differentiation potential of NDPSCs, SHED, and DPSCs. ((a1), (b1), and (c1)). Adipogenic differentiation is visually marked by accumulation of neutral lipid vacuoles in cultures (Oil Red staining). Actin ((a1) and (b1)) and vimentin (c1) expression were shown in green and nuclei in blue with DAPI. ((a2), (b2), and (c2)) Osteogenic differentiation was indicated by the formation of calcified nodule with Alizarin Red S staining. ((a3), (b3), and (c3)) The analyzed sections were positive for Safranin O staining after chondrogenic differentiation.

Measurement of differentiation potential is required to assess multipotential characteristics of stem cells. Histochemical and immunofluorescence methods were used to demonstrate differentiation potentials of each cell line into adipogenic, osteogenic, and chondrogenic states. During adipogenic differentiation, the lipid droplets enlarged and invaded the entire cytoplasm (Figure 4. (a1), (b1), and (c1)) while during osteogenic differentiation calcium deposits were clearly identified by Alizarin Red staining (Figure 4. (a2), (b2), and (c2)). Chondrogenic differentiation was characterized by the presence of round cells which resembled hyalin chondrocytes. The presence of GAG was shown by Safranin O staining (Figure 4. (a3), (b3), and (c3)). The same staining procedures were performed with the undifferentiated stem cells and were presented as negative staining in Supplementary Figure 2. 

**Figure 5:**
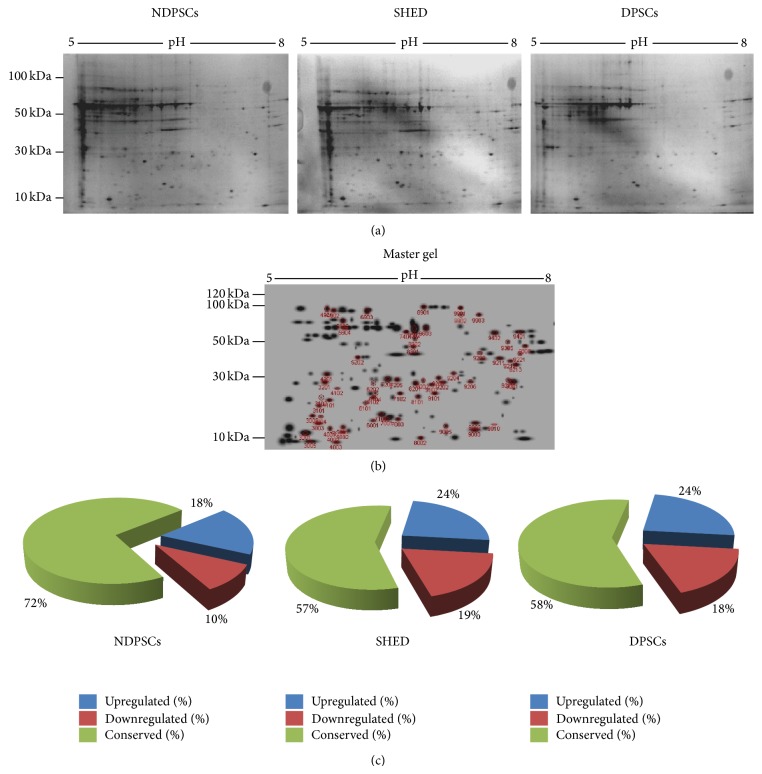
Comparative proteome analysis of NDPSCs, SHED, and DPSCs. (a) Undifferentiated MSCs were subjected to protein isolation and loaded onto pH 5 to pH 8 IPG strips for the first dimension and precast SDS-PAGE gels for the second dimension separation and stained with Colloidal Coomassie Blue for 24 hr after 24 hours of fixation. The gels are representative of three gels from each MSC type. (b) Master gel image was created by PDQuest Advance software to determine the number of spots that matched every member (183 spots). The numbers represent SSP numbers assigned to each spot by the software. These spots were cut from the gels and were subjected to MALDI-TOF/TOF analysis. Protein identification was performed by peptide mass finger printing by MASCOT (c) Pie charts to illustrate proteome conservation ratios among hNDP, SHED, and hDP-MSCs. Changes in spot intensities among 183 matching spots were compared. Spots that were up- or downregulated more than 2-fold were considered to be subjected to regulation.

A comparative proteomic analysis was performed to identify protein spots that were commonly expressed among the dental pulp derived MSCs. For this purpose stem cells were grown in defined media and protein samples from each MSC type were solubilized and separated by using IPG strips and SDS-PAGE. Following Colloidal Coomassie staining, well resolved and reproducible 2DE gel maps were produced as shown in Figure 5.

An average of 560 ± 30 well stained spots per analytical gel were detected when the gels were subjected to automated spot detection and analysis. However, the number of well-resolved spots that matched every member was 183 (Master Gel) with an overall mean coefficient of 60.8 percent. The rest of the spots were either not matchable or not expressed by all three stem cell types. By using PDQuest Advance gel analysis software, changes in spot intensities among these 183 matching spots were compared. Spots that were up- or downregulated more than 2-fold were considered to be subjected to regulation (Supplementary Figure 1). We found that 62.3 ± 7% of the protein spots were conserved. Ratios for up- and downregulated protein spots were given in Figure 5. In overall, analysis of spot scattering plots and conservation scores indicated that NDPSCs cell proteome was more similar to the SHED proteome than the DPSCs proteome.

**Figure 6:**
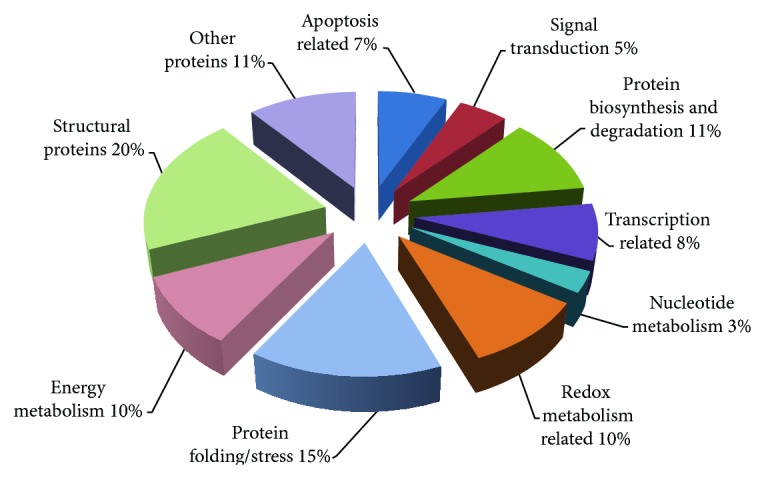
Classification of identified proteins based on their molecular function and their involvement in biological processes. Pie chart representing the distribution of the 61 identified proteins based on their molecular function and biological processes. Assignments were made on the basis of information from PANTHER analysis (http://www.pantherdb.org/) as well as NCBI (http://www.ncbi.nlm.nih.gov/pubmed) and Swiss-Prot/TrEMBL annotations (http://www.expasy.org/).

To further characterize conserved protein spots, the spots were cut from a preparative gel with an automated spot cutting instrument and subjected to in-gel tryptic digestion followed by MALDI-TOF/TOF analysis. Collectively, 61 protein spots were identified (Table 2). The identified proteins were subjected to analysis based on their molecular function and their involvement in biological processes (Table 3) (Figure 6).

**Figure 7:**
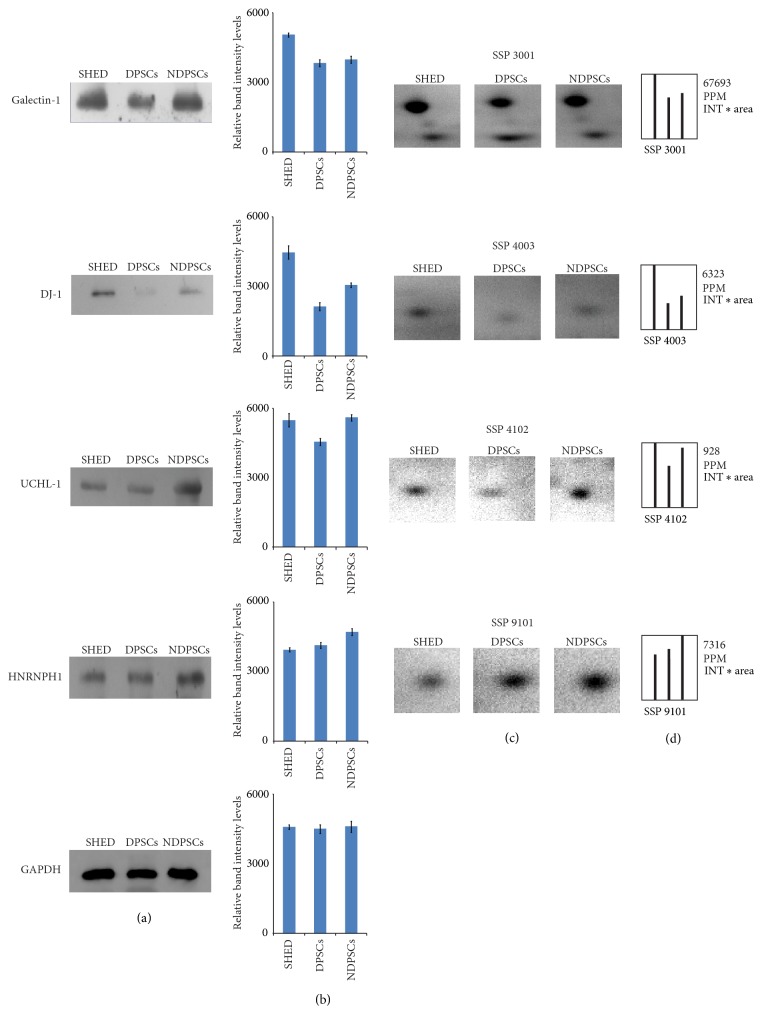
WB validation of the selected proteins identified by MALDI-TOF/TOF analysis. Western blot analysis was used for validation of proteomic results. Conventional 12% SDS gels were run with whole cell extracts. (a) Three lanes for SHED, DPSCs, and NDP-MSC are shown for each antibody. Galectin-1, DJ-1, UCHL-1, and HNRNPH1 antibodies were used for validation of expressions of the identified proteins. GAPDH was used for the normalization of each protein sample. (b) Relative protein intensities of bands were measured by Quantity One 1D analysis software (Bio-Rad). Each WB was repeated for three times. (c) Representative images of protein spots selected for WB analysis (protein identities corresponding to SSP numbers can be found in Table 2). (d) Comparative intensity graphs of selected protein spots generated by PDQuest Advance software (Bio-Rad).

To provide evidence to prove that the results of proteomic analysis were reliable, WB analysis for selected proteins was performed (Figure 7). The results agreed with the proteome analysis indicating that the observed changes in protein spots on 2DE gels were reliable and real.

Most of the identified proteins were shown to play roles in cellular architecture. Also, proteins that are part of protein folding machinery were described. Some of these proteins are inducible chaperons that are expressed under stress conditions. Transcription, protein biosynthesis, and degradation related proteins that we detected indicated the presence of active cell growth as predicted from cell cycle experiments. Redox metabolism related scavenger proteins that were detected may be an indicator of active cell growth as well. The presence of apoptosis, transcription, protein biosynthesis/degradation, and energy metabolism related proteins indicated the presence of cellular self-renewal and proliferation.

## 4. Discussion

In the presented study, we attempted to compare multilineage potentials and proteomic profiles of dental pulp derived MSCs isolated from dental pulps of three donors. Our aim was to minimize sample complexity by keeping sample number at three. One might argue that using more material from more donors might produce much more scientifically relevant data. However, increasing the number of donors makes analysis more complicated as each donor has different metabolic status (age, smoking, obesity, metabolic diseases, ethnicity, gender, and undetermined/unknown conditions). Indeed, our initial trials of proteome comparisons using samples from multiple donors generated more variable data and as the number of donors increased in groups the proteome profiles started to deviate more. Therefore, although there are examples in the literature which used samples from multiple donors [12–14], we preferred to use single donor for each group.

The MSCs were compared on the basis of their morphological properties, proliferation rates, expression of common MSC markers, and* in vitro* differentiation potentials into adipocytes, osteoblasts, and chondrocytes. Telomerase activities were also measured to monitor the proliferative history of each cell type. Finally, proteome analysis was performed by 2DE gel electrophoresis with MALDI-TOF/TOF MS/MS to create a protein expression pattern to help with understanding of metabolic pathways that were shared among these MSCs. For proteomic characterization experiments, a better controlled environment was created by using defined media.

In this study, we demonstrated that similarly isolated and grown MSCs from human dental pulp displayed similar morphology, antigenic phenotype, and differentiation potential. These findings do not differ from the previous findings reported by the other studies [12, 15–17]. However, in this study, we demonstrated that similarly isolated and grown MSCs from dental pulps of three different individuals carried considerable variation in their proteomes. A recent study reported similar findings among the proteomes of MSCs isolated from dental tissues (follicle, pulp, and papilla tissue) of a single donor [12]. They reported an average protein spot match ratio of 65% among the isolated MSCs. We also found low spot conservation scores even though we took an extreme precaution during cell culture and 2DE gel electrophoresis to prevent experimental variations. The precautions included the following. (I) Stem cells from three different dental pulps were isolated by using the same stem cell isolation protocol. (II) Cells were grown in defined growth media under the same growth conditions. (III) Before each passage, cells were allowed to grow to 70% confluency and equal numbers of cells were used to start new cultures. (IV) To prevent hand to hand variation, the same researchers performed the isolation, growth, and passaging experiments. (V) Protein isolation and 2DE experiments were also performed by a group of the same researchers. (VI) To prevent gel to gel variations in 2DE experiments, precast gels were used. They ran and stained under the same conditions by using Criterion gel running and staining systems under appropriate conditions.

Sixty-one of the every-member matching protein spots were identified by MALDI-TOF/TOF MS/MS. The identified proteins were subjected to analysis based on their molecular function and their involvement in biological processes. A total of 10 groups can be formed (Figure 6). Classification of the identified proteins based on molecular function revealed that 11 of the proteins can be grouped as structurally important ones. Among them, actin related protein complex 2/3 regulates tight junctions and function in establishment of branched actin networks [18]. To the best of our knowledge, the existence of this protein has not been described in MSCs. Calponin, a thin filament-associated protein that is implicated in the regulation and modulation of smooth muscle contraction, was also expressed by all our stem cell types. The expression of calponin in human hair follicle derived stem cells and bone marrow derived stem cells was previously reported [19]. Calponin was occasionally used as a marker to characterize smooth muscle cell derived stem cells [20]. Caldesmon is an actin- and myosin-binding protein and cellular component of movement and muscle contraction [21]. There is a strong interaction between caldesmon and calponin [22]. The identification of caldesmon and calponin in our stem cell populations may indicate that certain subpopulations of stem cells in our cultures might be committed to vascular smooth muscle lineage. However, the expression of actin interactin proteins may be due to well known motile nature of MSCs. In addition, expression of caldesmon and annexin A1 is proposed to be a marker for slow growth rate [23]. Because our stem cells were in defined medium their growth rates are expected to be slow and thus they might have expressed these two proteins at a respectable level. Zyxin binds alpha-actinin and the CRP protein and helps cell adhesion [24] and the cells used in this study expressed Zyxin. Zyxin was also determined to be expressed in hMSCs and proposed to be involved in cell to matrix contacts [25]. Previously, coactosin-like protein is shown to be highly expressed in amniotic-fluid derived MSCs [26]. Coactosin-like protein is involved in cytoskeletal organization and movement by binding actin. Both Coactosin-like protein and actin expression were identified in this study.

We have identified five proteins that have roles in redox metabolism and detoxification of the cells. Among them, glutathione S-transferase-P is known to function in detoxification of cells from xenobiotics and decrease susceptibility to cancer. In a previous study, glutathione S-transferase-P is used to distinguish multipotent MSCs isolated from bone marrow [27]. Observation of expression of glutathione S-transferase-P in our stem cells may indicate that our cells did have multipotent MSC properties.

Increased generation of reactive oxygen species (ROS) may be associated with differentiation process. On that line of thought, Clair et al. (1994) demonstrated the expression of MnSOD on cellular differentiation of fibroblasts [28]. They found that MnSOD greatly enhanced fibroblast differentiation into myoblasts. In addition, the protective effect of SOD was demonstrated in bone marrow derived stem cells and in hematopoietic stem cells [29, 30]. We also detected SOD expression in our stem cells. In addition to SOD, three other proteins that are involved in clearance of ROS were detected in this study. These were peroxiredoxin-2, thioredoxin domain-containing protein 12, and thioredoxin-dependent peroxide reductase. Peroxiredoxin reduces peroxides with reducing equivalents provided through the thioredoxin system. The expression of proteins displaying antioxidant activities by MSCs was previously recognized [31]. In fact MSCs were proposed to be used as a tool to clear out ROS from niches that are under oxidative stress [32]. We believe that all four enzymes that we report here protect cells from antioxidants and may have proliferative effects by eliminating peroxides.

Majority of the proteins that we identified have roles in protein folding machinery. For instance, peptidyl-prolyl* cis-trans* isomerase (PIN1) accelerates the folding of proteins by catalyzing* cis-trans* isomerization of proline imidic bonds in oligopeptides [33]. The role of PIN1 in odontogenic and adipogenic differentiation in human dental pulp derived stem cells was studied [34]. It was shown that PIN1 acted as an important modulator of odontogenic and adipogenic differentiation of DPSCs. PIN1 was also shown to promote survival, enhance repair, improve differentiation, and antagonize senescence [35]. PIN1 mRNA and protein levels were upregulated in a time-dependent manner during adipogenic differentiation in human dental pulp derived stem cells [34]. PIN1 may act as an important modulator in human dental pulp derived stem cells and may have significant implications in regenerative medicine. In a study by Nakamura et al. (2012), depression of PIN1 has shown to suppress neuronal differentiation while its overexpression enhanced it [36]. Our group has demonstrated that human dental pulp stem cells display better neural and epithelial stem cell properties [37]. A distinct role for PIN1 has been assigned in induction and maintenance of pluripotency [38]. PIN1 was described to be an indispensable factor for the self-renewal and maintenance of pluripotent stem cells because it can regulate Oct4 and other substrates via activation of phosphorylation cascades. Therefore, the presence of PIN1 in our stem cells may be related to stemness properties of human dental pulp derived stem cells.

There were four heat shock proteins (60 kDa heat shock protein, heat shock cognate 71 kDa protein, heat shock protein Beta-1, and 78 kDa glucose-regulated protein) that we identified and they were expressed by all three stem cells. Inducible and constitutive HSP proteins confer synergetic resistance against metabolic changes [39]. Protein DJ-1 is another chaperone protein that protects cells against oxidative stress and cell death. Its role in Parkinson's disease is well known but its significance in stem cell research has not been studied [40]. UPF0556 protein C19orf10 (interleukin-25) is another protein that we detected in our gels. This protein is a secreted protein and is proposed to activate signaling pathways that is involved in unfolded protein response [41]. The last protein that we detected and plays a role in protein folding was tubulin-specific chaperone A which is involved in early steps of the tubulin folding pathway [42]. The presence of chaperons or proteins that play roles in protein folding machinery may indicate the efforts of preservation of stem cell integrity.

We identified several proteins that play roles in transcription and nucleotide metabolism. Among them, PSPC1 is interesting since the functions of paraspeckles in eukaryotic cells are still not known. The presence of a paraspeckle protein (PSP1 alpha) was reported in human embryonic stem cells although the existence of paraspeckles in human embryonic stem cells was reported to occur only upon differentiation [43]. Our cells expressed a paraspeckle component, PSP1C, and this suggested that paraspeckle components are not only expressed by human embryonic stem cells but also expressed by human mesenchymal stem cells and their expression may not require a differentiation process.

Twenty percent of the identified proteins displayed binding activities and five of these proteins are involved in calcium or calcium binding metabolism (protein S100-A6, annexin A1, protein S100-A11, peptidyl-prolyl* cis-trans* isomerase, and gelsolin). The importance of calcium metabolism has long been recognized in stem cells. In fact, calcium hydroxide is shown to increase recruitment, migration, proliferation, and mineralization of the dental pulp derived MSCs [44]. In a recent study, Chen et al. (2013) demonstrated the effect of S100A4, another calcium binding protein, on proliferation, survival, and differentiation of human osteosarcoma cells [45]. In a more recent study, SA100A6 is proposed to be a novel marker for neural stem cells and may be important for generation of astrocytes in the adult hippocampus [46]. Annexin, which interacts with SA100A6, is required for midbody formation and completion of the terminal phase of cytokinesis and was identified in our study as a part of the calcium binding metabolism.

The presence of four apoptosis related proteins indicated that self-renewal and proliferation of stem cell populations is controlled by induction of apoptosis. Therefore, there is always a balance between the number of actively growing stem cells and apoptotic stem cells. In overall, the dynamic nature of stem cell populations is evident by the presence of apoptosis related proteins as well as proteins that are involved in protein biosynthesis/degradation, energy metabolism, and transcription.

## 5. Conclusions

In this study we used basic cell biology and proteomics techniques to uncover the heterogeneity of proteins and their characteristics among three stem cell types. The cells displayed similar features related to morphology, proliferation rates, expression of various cell surface markers, and differentiation potentials. Furthermore, using 2DE with MALDI-TOF/TOF MS/MS approach, we have generated a proteomic profile. We mainly focused on the 61 proteins that were predominantly expressed by all three stem cell types. We are aware that other differentially expressed proteins in each cell type may also be important in understanding specific proteins representing three MSCs from different age groups and those proteins may be a subject of another study. Some of the identified proteins in this study may hold importance in understanding specific properties of human dental pulp derived mesenchymal stem cells. Clarification of the similarities and differences among the proteomes of human dental pulp derived mesenchymal stem cells will help to have a better understanding of these cells and this study is a step taken towards that direction.

## Supplementary Material

Supplementary Figure 1 is for selected representative protein spots that were subjected to regulation among every-member-matching spots. Supplementary Figure 2 is composed of images representing negative staining profiles for NDPSCs, SHED and DPSCs.

## Figures and Tables

**Table 1:** Immunophenotypic characteristics of NDPSCs, SHED, and hDDP-MSCs. The figures in the table are percentages of CD positive cells.

Markers	NDPSCs	SHED	DPSCs
CD13	90.87 ± 5.24	89.73 ± 6.76	94.34 ± 8.42
CD14	0.78 ± 0.62	0.78 ± 0.86	0.16 ± 0.07
CD29	99.43 ± 0.28	99.88 ± 0.08	99.44 ± 0.37
CD34	0.93 ± 0.62	0.24 ± 0.08	0.29 ± 0.24
CD44	98.62 ± 1.33	99.80 ± 0.12	99.77 ± 0.26
CD45	0.65 ± 0.42	0.28 ± 0.09	0.12 ± 0.05
CD73	99.69 ± 0.36	99.76 ± 0.36	99.56 ± 0.14
CD90	97.62 ± 3.17	99.73 ± 0.28	99.49 ± 0.32
CD106	1.04 ± 0.70	1.18 ± 0.39	0.14 ± 0.02
CD117	5.68 ± 0.24	1.74 ± 1.72	0.94 ± 0.85
CD146	81.38 ± 7.33	57.91 ± 0.76	54.44 ± 23.89
CD166	99.15 ± 0.97	99.51 ± 0.60	98.59 ± 0.97
HLA-ABC	94.88 ± 5.25	66.89 ± 6.36	83.15 ± 5.65
HLA-DR	0.56 ± 0.39	0.12 ± 0.02	0.17 ± 0.05

**Table 2:** Protein spots that were identified by MALDI-TOF/TOF (MS/MS) analysis.

SSP	Swiss-Prot accession	Best protein description	Sequence coverage/matches	MASCOT probability/expect (p)	Theoretical MW (Da)	Experimental p*I*
3001	P 09382	Galectin-1	40%/9 fragment	236/5.10*e − *20	14706	5.34
3005	P 06703	Protein S100-A6	24%/7 fragment	124/8.10*e − *09	10173	5.33
4003	P 62988	Ubiquitin	31%/5 fragment	151/1.60*e − *11	8560	6.56
5002	O 75368	SH3 domain-binding glutamic acid-rich-like protein	22%/5 fragment	181/1.60*e − *14	12766	5.22
5001	Q 14019	Coactosin-like protein	28%/7 fragment	93/1.00*e − *05	15935	5.54
4001	O 75347	Tubulin-specific chaperone A	48%/10 fragment	295/6.40*e − *26	12847	5.25
3003	Q 9NQ39	Putative 40S ribosomal protein S10-like	20%/7 fragment	22/1.10*e* + 02	20108	10.13
3002	P 63241	Eukaryotic translation initiation factor 5A-1	15%/5 fragment	177/4.00*e − *14	16821	5.08
3004	O 95881	Thioredoxin domain-containing protein 12	12%/4 fragment	47/0.41	19194	5.24
3101	P 52815	39S ribosomal protein L12, mitochondrial	15%/4 fragment	164/8.10*e − *13	21335	9.04
4101	O 75947	ATP synthase subunit d, mitochondrial	32%/8 fragment	322/1.30*e − *28	18480	5.21
4102	P 09936	Ubiquitin carboxyl-terminal hydrolase isozyme L1	22%/8 fragment	212/1.30*e − *17	24808	5.33
4002	P 07858	Cathepsin B	5%/1 fragment	107/4.00*e − *07	37797	5.88
3201	P 43487	Ran-specific GTPase-activating protein	28%/10 fragment	167/4.00*e − *13	23296	5.19
6102	P 32119	Peroxiredoxin-2	34%/15 fragment	536/5.10*e − *50	21878	5.66
6104	P 30085	UMP-CMP kinase	13%/5 fragment	140/2.00*e − *10	22208	5.44
6202∗	P 04792	Heat shock protein beta-1	33%/12 fragment	321/1.60*e − *28	22768	5.98
7101	P 00441	Superoxide dismutase [Cu-Zn]	9%/2 fragment	101/1.60*e − *06	15926	5.7
6001	O 15511	Actin related protein 2/3 complex subunit 5	12%/3 fragment	78/0.00035	16310	5.47
7001	Q 12912	Lymphoid-restricted membrane protein	1%/2 fragment	25/67	62069	5.62
7204	P 07339	Cathepsin D	20%/17 fragment	503/1.00*e − *46	44524	6.1
7205	Q 99436	Proteasome subunit beta type 7	14%/9 fragment	150/2.00*e − *11	29946	7.57
7102	P 09211	Glutathione S-transferase P	16%/5 fragment	215/6.4*e − *018	23341	5.43
4201A∗	P 07858	Cathepsin B	28%/14 fragment	574/8.10*e − *54	37797	5.88
4201B	O 00299	Chloride intracellular channel protein 1	10%/6 fragment	130/2.00*e − *09	26906	5.09
8101	P 04792	Heat shock protein beta-1	45%/22 fragment	707/4.00*e − *67	22768	5.98
8201	P 30048	Thioredoxin-dependent peroxide reductase, mitochondrial	23%/10 fragment	346/5.10*e − *31	27675	7.67
9102	O 95571	Protein ETHE1, mitochondrial	35%/12 fragment	439/2.60*e − *40	27855	6.35
9101	Q 99497	Protein DJ-1	33%/10 fragment	239/2.60*e − *20	19878	6.33
8202	P 30084	Enoyl-CoA hydratase, mitochondrial	25%/10 fragment	183/1.00*e − *14	31367	8.34
9202∗	P 60174	Triosephosphate isomerase	64%/24 fragment	872/1.30*e − *83	26653	6.45
9203∗	P 07339	Cathepsin D	16%/14 fragment	233/1.00*e − *19	44524	6.1
8002	P 31949	Protein S100-A11	56%/10 fragment	312/1.30*e − *27	11733	6.56
9003	P 49773	Histidine triad nucleotide-binding protein 1	16%/4 fragment	139/2.60*e − *10	13793	6.43
9002	Q 969H8	UPF0556 protein C19orf10	30%/12 fragment	437/4.00*e − *40	18783	6.2
9201	P 60174	Triosephosphate isomerase	71%/28 fragment	994/8.10*e − *96	26653	6.45
9215	P 18669	Phosphoglycerate mutase 1	41%/13 fragment	627/4.00*e − *59	28786	6.67
9213	O 00560	Syntenin-1	24%/13 fragment	257/4.00*e − *22	32424	7.05
9212	Q 99439	Calponin-2	11%/9 fragment	79/0.00027	33675	6.95
9211	O 14979	Heterogeneous nuclear ribonucleoprotein D-like	15%/13 fragment	361/1.60*e − *32	46409	9.59
9209	P 04083	Annexin A1	15%/9 fragment	233/1.00*e − *19	38690	6.57
9221	Q 99729	Heterogeneous nuclear ribonucleoprotein A/B	18%/8 fragment	145/6.40*e − *11	36202	8.22
9308	Q 14103	Heterogeneous nuclear ribonucleoprotein D0	20%/11 fragment	301/1.60*e − *26	38410	7.62
9305	P 60709	Actin, cytoplasmic 1	24%/14 fragment	360/2.00*e − *32	41710	5.29
9402	P 06733	Alpha-enolase	24%/19 fragment	494/8.10*e − *46	47139	7.01
9401	P 13929	Beta-enolase	14%/8 fragment	165/6.40*e − *13	46902	7.59
9401	P 09104	Gamma-enolase	11%/6 fragment	159/2.60*e − *12	47239	4.91
9903	Q 8WXF1	Paraspeckle component 1	17%/14 fragment	236/5.10*e − *20	58706	6.26
9902	P 04264	Keratin, type II cytoskeletal 1	9%/9 fragment	416/5.10*e − *38	65999	8.15
9901	Q 05682	Caldesmon	24%/23 fragment	512/1.30*e − *47	93194	5.63
8901	Q 15942	Zyxin	21%/19 fragment	287/4.00*e − *25	61238	6.22
8502	P 17661	Desmin	14%/10 fragment	137/4.00*e − *10	53503	5.21
7401	P 31943	Heterogeneous nuclear ribonucleoprotein H	49%/29 fragment	966/5.10*e − *93	49198	5.89
9603	P 08670	Vimentin	75%/57 fragment	1290/2.00*e − *125	53619	5.06
8302	O 14773	Tripeptidyl-peptidase 1	4%/4 fragment	189/2.60*e − *15	61210	6.01
8301	P 40121	Macrophage-capping protein	23%/18 fragment	465/6.40*e − *43	38494	5.88
5202	P 52907	F-actin-capping protein subunit alpha-1	44%/11 fragment	210/2.00*e − *17	32902	5.45
6903	P 11142	Heat shock cognate 71 kDa protein	24%/19 fragment	472/1.3*e − *043	70854	5.37
5902	P 10809	60 kDa heat shock protein, mitochondrial	27%/18 fragment	475/6.4*e − *044	61016	5.7
5604	Q 16352	Alpha-internexin	5%/3 fragment	72/0.0012	55357	5.34
4902∗	Q 96AY3	Peptidyl-prolyl *cis-trans* isomerase FKBP10	5%/5 fragment	73/0.00093	64204	5.36
4901	P 11021	78 kDa glucose-regulated protein	22%/16 fragment	426/5.1*e − *039	72288	5.07
7003	P 15531	Nucleoside diphosphate kinase A	37%/11 fragment	348/3.20*e − *31	17138	5.83
9005	Q 01469	Fatty acid-binding protein	27%/5 fragment	246/5.10*e − *21	15155	6.6
9204	P 25786	Proteasome subunit alpha type 1	25%/9 fragment	166/5.1*e − *013	29537	6.15
9206∗	P 60174	Triosephosphate isomerase	30%/8 fragment	116/5.1*e − *008	26653	6.45
9010	P 62937	Peptidyl-prolyl *cis-trans* isomerase A	17%/5 fragment	150/2.00*e − *11	18001	7.68

^*^Some of the identified proteins appeared as more than one spot on the gels as indicated by the presence of more than one SSP number.

**Table 3:** Classification of the proteins that were identified by MALDI-TOF/TOF analysis. The classifications were made based on PANTHER analysis and Swiss-Prot annotations.

Protein name	Function	Biological process involved
Apoptosis related		
Galectin	Apoptosis, cell proliferation, and differentiation	Apoptosis
Cathepsin B	Thiol protease which is believed to participate in intracellular degradation and turnover of proteins.	Regulation of apoptotic process and proteolysis
Cathepsin D	Acid protease active in intracellular protein breakdown	Regulation of apoptotic process and proteolysis
Histidine triad nucleotide-binding protein 1	Hydrolyzes purine nucleotide phosphoramidates	Apoptosis

Signal transduction		
SA100A6	May function as calcium sensor and modulator, contributing to cellular calcium signaling	Signal transduction
Protein S100-A11		Signal transduction
Annexin A1	Calcium/phospholipid-binding protein which promotes membrane fusion and is involved in exocytosis	Cell surface receptor signaling pathway

Protein biosynthesis and degradation		
Ubiquitin	Behaves as a molecular tag for degradation of proteins	Ubiquitination
Putative 40S ribosomal protein S10-like	Ribonucleoprotein	Protein biosynthesis
Eukaryotic translation initiation factor 5A-1	Ribosome binding Translation elongation factor activity	Protein biosynthesis
Ubiquitin carboxyl-terminal hydrolase	Processing of ubiquitin precursors and of ubiquitinated proteins	Ubl conjugation pathway
39S ribosomal protein L12, mitochondrial	Ribonucleoprotein	Mitochondrial protein biosynthesis
Proteasome subunit alpha type 1	The proteasome is a multicatalytic proteinase complex which is characterized by its ability to cleave peptides with Arg, Phe, Tyr, Leu, and Glu adjacent to the leaving group at neutral or slightly basic pH	Immunity
Proteasome subunit beta type 7	Cleavage of peptide bonds with very broad specificity	Protein degradation

Transcription related		
Heterogeneous nuclear ribonucleoprotein A/B	Binds single-stranded RNA. Has a high affinity for G-rich and U-rich regions of hnRNA	Transcription, transcription regulation
Heterogeneous nuclear ribonucleoprotein D0	Binds with high affinity to RNA molecules that contain AU-rich elements (AREs) found within the 3′-UTR of many protooncogenes and cytokine mRNAs	Transcription, transcription regulation
Heterogeneous nuclear ribonucleoprotein H	This protein is a component of the heterogeneous nuclear ribonucleoprotein (hnRNP) complexes which provide the substrate for the processing events that pre-mRNAs undergo before becoming functional, translatable mRNAs in the cytoplasm. Mediates pre-mRNA alternative splicing regulation	Mediates pre-mRNA alternative splicing regulation
Heterogeneous nuclear ribonucleoprotein D-like	Acts as a transcriptional regulator. Promotes transcription repression.	Transcription, transcription regulation
Paraspeckle component 1	Regulates, cooperatively with NONO and SFPQ, androgen receptor-mediated gene transcription activity in Sertoli cell line	Transcription, transcription regulation

Nucleotide metabolism		
UMP-CMP kinase	Catalyzes the phosphorylation of pyrimidine nucleoside monophosphates at the expense of ATP	Nucleotide metabolism
Nucleoside diphosphate kinase A	Major role in the synthesis of nucleoside triphosphates other than ATP	Nucleotide metabolism

Redox metabolism related		
Thioredoxin domain-containing protein 12	Possesses significant protein thiol-disulfide oxidase activity	Oxidoreductase
Peroxiredoxin-2	Involved in redox regulation of the cell. Reduces peroxides with reducing equivalents provided through the thioredoxin system	Hydrogen peroxide catabolic process removal of superoxide radicals
Superoxide dismutase [Cu-Zn]	Destroys radicals which are normally produced within the cells and which are toxic to biological systems.	Antioxidant, oxidoreductase
Thioredoxin-dependent peroxide reductase	Peroxidase activity, peroxiredoxin activity	Involved in redox regulation of the cell
Glutathione S-transferase P	Conjugation of reduced glutathione to a wide number of exogenous and endogenous substrates	Glutathione derivative biosynthetic process
Persulfide dioxygenase ETHE1, mitochondrial	Plays ab essential role in hydrogen sulfide catabolism in the mitochondrial matrix	Hydrogen sulfide metabolic process

Protein folding/stress		
Tubulin-specific chaperone A	Involves the early step of the tubulin folding pathway.	Chaperone binding
UPF0556 protein C19orf10		Involved in unfolded protein response, cellular protein metabolic process
Protein DJ-1	Protects cells against oxidative stress and cell death.	Oxidative stress
Heat shock protein beta-1	Involved in stress resistance and actin organization.	Stress response
Heat shock cognate 71 kDa protein	Acts as a repressor of transcriptional activation	Stress response
60 kDa heat shock protein, mitochondrial	Implicated in mitochondrial protein import and macromolecular assembly	de novo' protein folding, stress response
78 kDa glucose-regulated protein	Plays a role in facilitating the assembly of multimeric protein complexes inside the endoplasmic reticulum	ER overload response
Peptidyl-prolyl *cis-trans* isomerase A	It catalyzes the *cis-trans* isomerization of proline imidic peptide bonds in oligopeptides.	PPIases accelerate the folding of proteins
Tripeptidyl-peptidase 1	Lysosomal serine protease with tripeptidyl-peptidase I activity	Involved in unfolded protein response

Energy metabolism		
ATP synthase subunit D	ATP synthesis	Energy metabolism
Phosphoglycerate mutase 1	Interconversion of 3- and 2-phosphoglycerate	Glycolysis, gluconeogenesis
Alpha-enolase	Role in glycolysis, plays a part in various processes such as growth control, hypoxia tolerance, and allergic responses.	Glycolysis, plasminogen activation Transcription, transcription regulation
Beta-enolase	Striated muscle development and regeneration.	Glycolysis
Gamma-enolase	Has neurotrophic and neuroprotective properties on a broad spectrum of central nervous system (CNS) neurons	Glycolysis
Triosephosphate isomerase	D-Glyceraldehyde 3-phosphate = glycerone phosphate.	Energy metabolism

Structural proteins		
Coactosin-like protein	Acts as a chaperone for ALOX5 (5LO), influencing both its stability and activity in leukotrienes synthesis.	Binds to F-actin in a calcium-independent manner
Actin related protein 2/3 complex subunit 5	Component of the Arp2/3 complex which is involved in regulation of actin polymerization	Structural constituent of cytoskeleton
Syntenin-1	An adapter protein, in adherens junctions may function to couple syndecans to cytoskeletal proteins or signaling components	Actin cytoskeleton organization
Calponin-2	Thin filament-associated protein that is implicated in the regulation and modulation of smooth muscle contra	Actin binding
Actin, cytoplasmic 1	Cell motility	Cell structure formation
Keratin, type II cytoskeletal 1	Regulate the activity of kinases such as PKC and SRC via binding to integrin beta-1 (ITB1) and the receptor of activated protein kinase C	Complement activation, lectin pathway, fibrinolysis, and regulation of angiogenesis
Caldesmon	Actin- and myosin-binding protein	Cellular component movement, muscle contraction
Zyxin	Adhesion plaque protein. Binds alpha-actinin and the CRP prot	Cell adhesion
Desmin	Class-III intermediate filaments found in muscle cells	Cytoskeleton organization, muscle filament sliding
Vimentin	Vimentins are class-III intermediate filaments found in various nonepithelial cells, especially mesenchymal c	Intermediate filament organization
Macrophage-capping protein	Calcium-sensitive protein which reversibly blocks the barbed ends of actin filaments but does not sever preformed actin filaments. May play an important role in macrophage function.	Barbed-end actin filament capping, cell projection assembly
F-actin-capping protein subunit alpha-1	F-actin-capping proteins bind in a Ca^2+^-independent manner to the fast growing ends of actin filaments	Actin cytoskeleton organization, actin filament capping, cellular component movement

Other proteins		
SH3 domain-binding glutamic acid-rich-like protein	SH3/SH2 adaptor activity	SH3-binding
Ran-specific GTPase-activating protein	Inhibits GTP exchange on Ran	Positive regulation of GTPase activity
Lymphoid-restricted membrane protein	Vesicle fusion	Vesicle targeting
Chloride intracellular channel protein 1	Can be inserted into membranes and form chloride ion channels.	Positive regulation of osteoblast differentiation
Enoyl-CoA hydratase	Straight-chain enoyl-CoA thioesters from C4 up to at least C16	Fatty acid metabolism
Alpha-internexin	Class-IV neuronal intermediate filament	Differentiation, neurogenesis
Fatty acid-binding protein	High specificity for fatty acids. Highest affinity for C18 chain length.	Transport

## Abbreviations

DP: Dental pulp derived
hNDP: Natal dental pulp derived
SHED: Stem cells from human exfoliated deciduous
MSCs: Mesenchymal stem cells
MALDI: Matrix assisted laser desorption ionization
TOF: Time of flight
WB: Western blot
EDTA: Ethylenediaminetetraacetic acid
TMB: 3,3′,5,5′-Tetramethylbenzidine dihydrochloride
RTA: Relative telomerase activity
CHAPS: 3-[(3-Cholamidopropyl) dimethylammonio]-1-propanesulfonate
DTT: Dithiothreitol
IPG: Immobilized pH gradient
MS: Mass spectrometry
SDS-PAGE: Sodium dodecyl sulfate-polyacrylamide gel electrophoresis.
