# Supplementary figures and images for: Phenotypic and Proteomic Characteristics of Human Dental Pulp Derived Mesenchymal Stem Cells from a Natal, an Exfoliated Deciduous, and an Impacted Third Molar Tooth
Source: Stem Cells Int. 2014 Oct 14;2014:457059. doi: 10.1155/2014/457059 (PMC4212660; doi:10.1155/2014/457059)

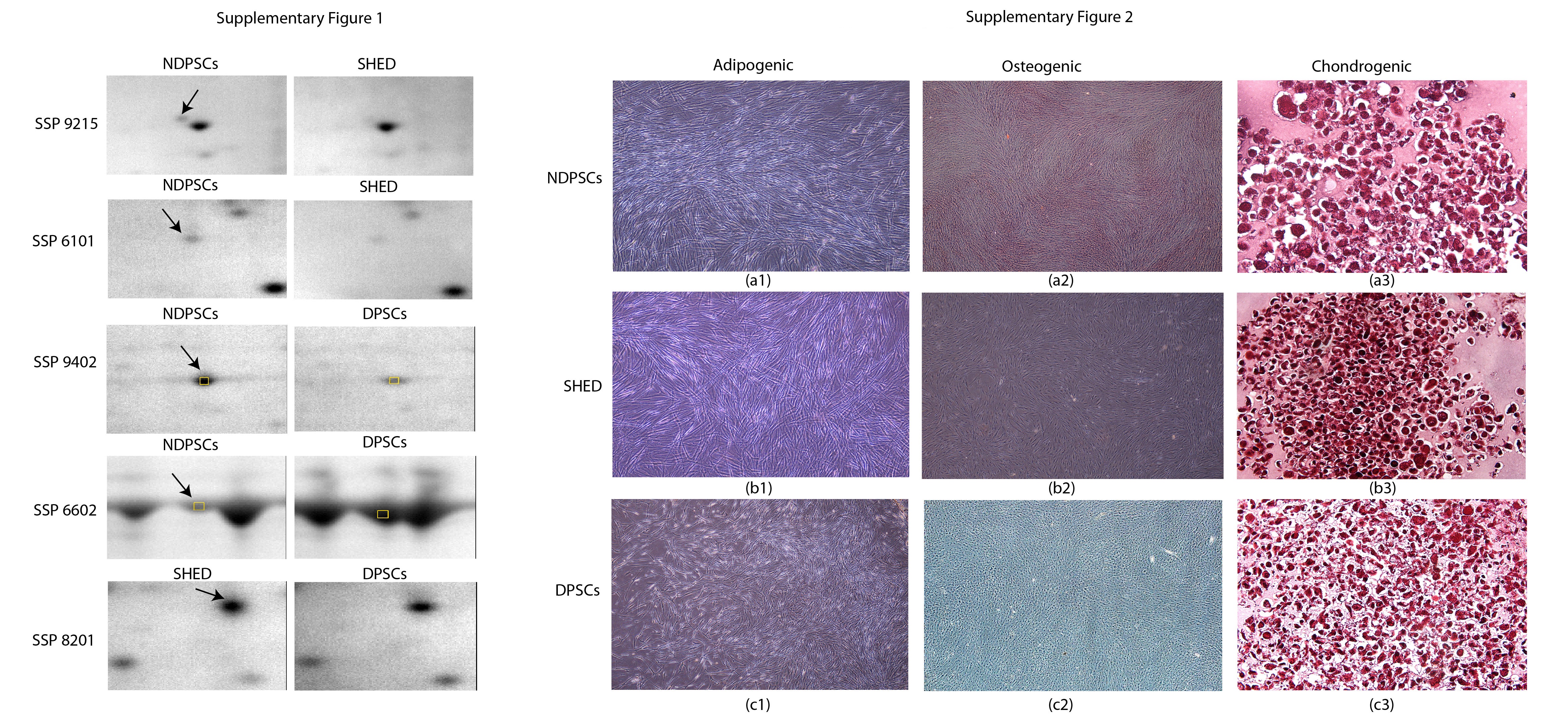

Supplement: Supplementary file 1 — Supplementary Figure 1 is for selected representative protein spots that were subjected to regulation among every-member-matching spots. Supplementary Figure 2 is composed of images representing negative staining profiles for NDPSCs, SHED and DPSCs. [file 457059.f1.jpg]
